# Annular leukocytoclastic vasculitis associated with anti-tuberculosis medications: a case report

**DOI:** 10.1186/1752-1947-7-34

**Published:** 2013-01-31

**Authors:** Kumutnart Chanprapaph, Wanjarus Roongpisuthipong, Kunlawat Thadanipon

**Affiliations:** 1 Division of Dermatology, Department of Medicine, Faculty of Medicine, Ramathibodi Hospital, Mahidol University, Bangkok, 10400Thailand; 2 Division of Dermatology, Department of Medicine, Faculty of Medicine, Vajira Hospital, University of Bangkok Metropolis, Bangkok, 10300Thailand

## Abstract

**Introduction:**

Anti-tuberculosis drug-induced cutaneous leukocytoclastic vasculitis has been rarely reported. To the best of our knowledge, this is the first reported case of annular leukocytoclastic vasculitis associated with anti-tuberculosis drug administration.

**Case presentation:**

We report a case of annular leukocytoclastic vasculitis induced by anti-tuberculosis medication. A 62-year-old Thai man presented to our facility with a generalized exanthematous rash on his trunk and extremities that resolved shortly afterwards. Subsequently, he developed multiple, erythematous-to-purplish, non-blanchable macules and papules with an annular arrangement on his extremities. The skin rash occurred after two weeks of anti-tuberculosis medication. The histopathology of the purpuric skin lesion was consistent with leukocytoclastic vasculitis. The skin lesion improved after discontinuation of the anti-tuberculosis drugs and treatment with oral antihistamine and topical corticosteroid drugs. Streptomycin, ethambutol and ofloxacin were administered as second-line anti-tuberculosis therapy during his hospitalization. No adverse reactions were observed.

**Conclusions:**

Leukocytoclastic vasculitis should be considered in the differential diagnosis of annular non-blanchable macules and papules. Although rare, anti-tuberculosis drugs should be considered potential causes of drug-induced annular leukocytoclastic vasculitis.

## Introduction

Leukocytoclastic vasculitis (LCV) is a histologically defined condition characterized by necrotizing inflammation around small dermal blood vessels, composed mainly of neutrophils and their debris. The usual presentation of LCV is polymorphous, with findings such as purpuric papules, urticaria and ulceration. An annular appearance of LCV is quite rare. Causes of LCV include drug reactions, malignancies, connective tissue diseases, infections and idiopathy [1,2]. An estimated 20 percent to 30 percent of all vasculitis cases are attributed to drug reactions. LCV has rarely been reported in association with anti-tuberculosis drugs [3]. We report an unusual clinical presentation of LCV following treatment with anti-tuberculosis drugs.

## Case presentation

**Figure 1:**
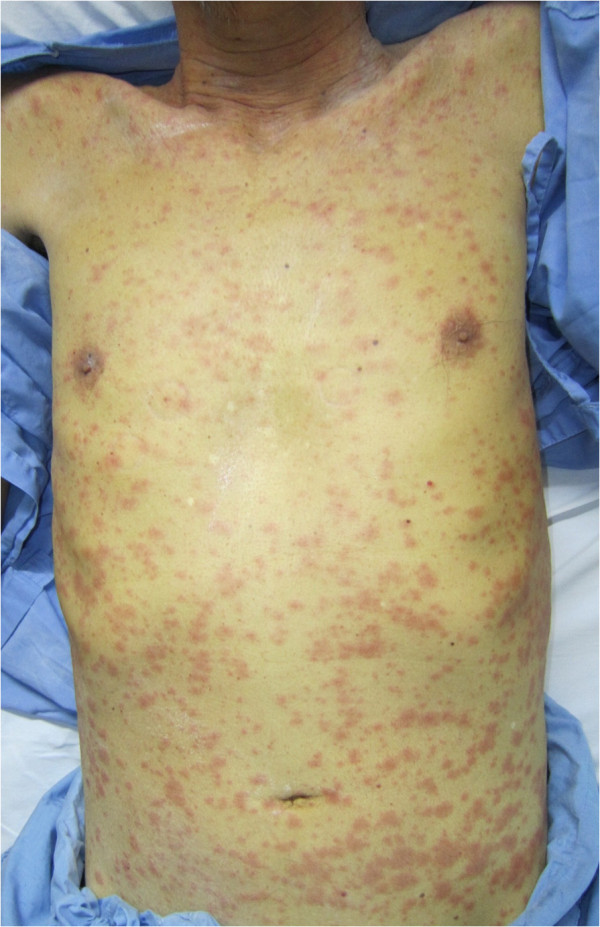
Generalized exanthematous rash on the trunk and extremities of our patient.

**Figure 2:**
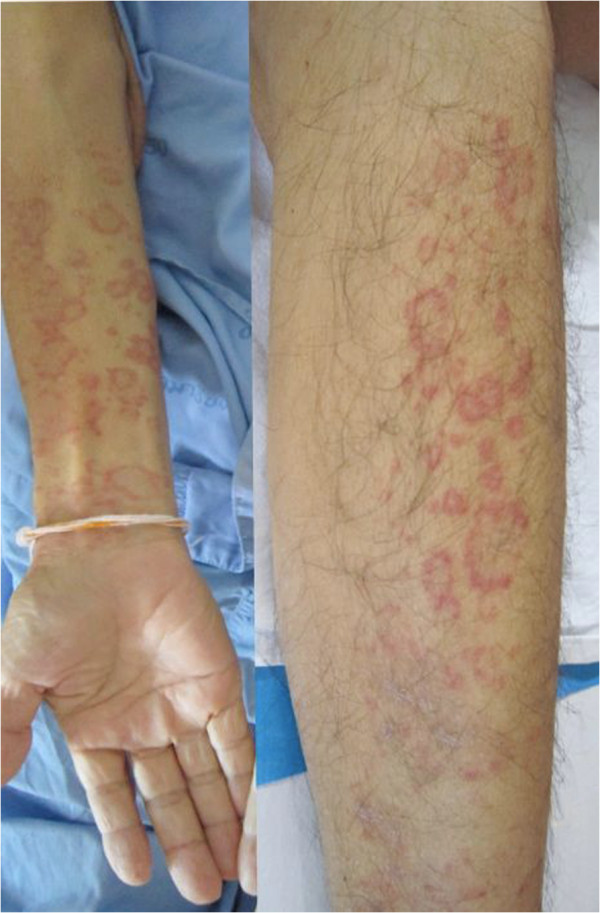
Multiple, well-defined, non-blanchable, erythematous macules and papules in an annular arrangement.

A 62-year-old Thai man presented to our facility with a generalized erythematous maculopapular rash on his trunk and extremities. Our patient had been diagnosed as having pulmonary tuberculosis two weeks earlier. He had long-standing hypertension and diabetes mellitus; his treatment for this included daily amlodipine 5mg, metformin 500mg and glipizide 5mg daily. When tuberculosis was diagnosed, anti-tuberculosis therapy of isoniazid 300mg, rifampicin 450mg and ethambutol 800mg daily was administered. After two weeks of anti-tuberculosis medication, a generalized exanthematous rash appeared on his trunk and extremities (Figure 1). All anti-tuberculosis drugs were stopped after his admission due to a clinical suspicion of drug-induced adverse cutaneous reactions. The exanthematous rash resolved within three days of admission, leaving post-inflammatory hyperpigmentation. One day later, multiple well-defined, non-blanchable, erythematous-to-purplish macules and papules, some of which showed an annular arrangement, were noticed (Figure 2). He had no history of drug allergy and a review of systems was unremarkable.

**Figure 3:**
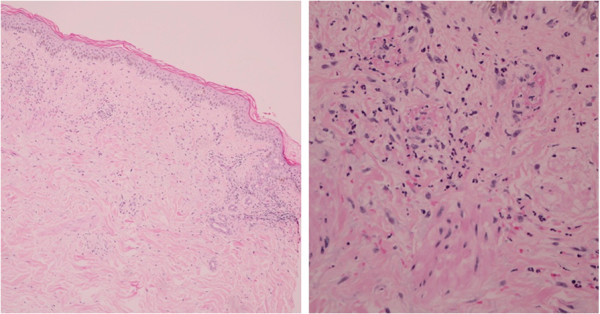
Histology of a purpuric annular lesion on our patient’s leg.

**Figure 4:**
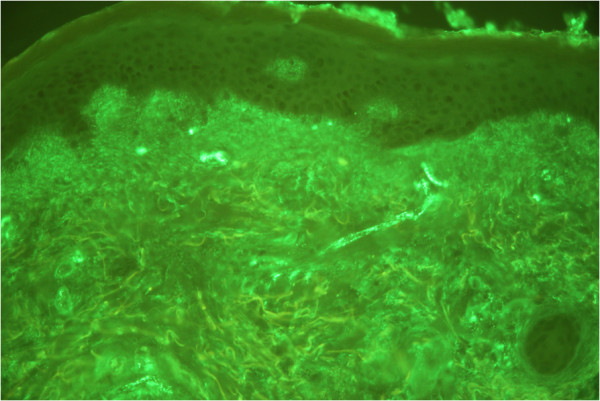
Direct immunofluorescence revealed positive IgA, IgM and C3 results along superficial dermal blood vessels.

Laboratory study findings were as follows: leukocytes, 12.8×10^3^ cells/mm^3^ (neutrophils 86 percent, lymphocytes 9 percent, monocytes 2 percent, eosinophils 2 percent, basophils 1 percent); hemoglobin, 7.9g/dL; platelet count, 215×10^3^ cells/mm^3^; creatinine, 1.2mg/dL; aspartate aminotransferase, 31U/L; alanine aminotransferase, 34U/L; alkaline phosphatase, 582U/L; γ-glutamyl transferase, 393U/L; total bilirubin, 9.6mg/dL; direct bilirubin, 8.4mg/dL; hepatitis B surface antigen, anti-hepatitis C virus and anti-human immunodeficiency virus (HIV) test results were all negative. Anti-nuclear antibody and anti-neutrophil cytoplasmic antibody test results were also negative. Urine analysis results showed no evidence of proteinuria or hematuria. The findings from an abdominal ultrasound were unremarkable. A skin biopsy from a purpuric annular lesion on his leg showed peri-vascular and interstitial infiltration of neutrophils with extravasation of erythrocytes and fibrin deposition (Figure 3), characteristic of LCV. A direct immunofluorescence (DIF) study was positive for IgA, IgM and C3 along superficial dermal blood vessels, consistent with cutaneous small vessel vasculitis (Figure 4). These findings were diagnostic for annular LCV associated with anti-tuberculosis drugs and drug-induced cholestasis. Our patient was treated with an oral antihistamine and topical corticosteroids. The skin eruption cleared within one week without hyperpigmentation and liver function test results returned to normal limits three weeks after the anti-tuberculosis drugs were withdrawn. Streptomycin (750mg per day), ethambutol (800mg per day) and ofloxacin (400mg per day) were subsequently administered as second-line anti-tuberculosis therapy during the hospitalization. No adverse reactions were observed. Therefore, he was subsequently treated with ethambutol, ofloxacin and streptomycin without recurrence of the skin rash.

## Discussion

Cutaneous adverse reaction to anti-tuberculosis drugs has been reported in up to 5 percent of patients treated. Common cutaneous reactions include pruritus, urticaria, maculopapular exanthems, fixed reaction and erythema nodosum. Cutaneous LCV is a rare complication of anti-tuberculosis medication [4]. The skin lesions typically improve upon withdrawal of the medication. In a previous study a case of rifampicin-induced and pyrazinamide-induced LCV was reported, and it was suggested that antibodies to drugs contribute to the pathogenesis of vasculitis [5]. Cribier *et al*. first reported that some patients with annular LCV constitute a distinctive subtype, with the following criteria [6]: (1) multiple attacks over years with sudden onset and spontaneous regression after seven to 10 days, (2) annular purpuric patches that show centrifugal extension, (3) extension over the limbs and trunk creating polycyclic lesions that clear leaving mild hemosiderin deposition, (4) no extracutaneous symptoms and good general health, (5) histological changes of LCV with mild vascular changes and intense polymorphonuclear cell infiltration, and (6) complete clearance of all lesions with dapsone (diamino-diphenyl sulfone). Our patient’s case matches the second, third, fourth and fifth criteria established by Cribier *et al*. Therefore, we cannot completely classify our patient’s case as a distinct subtype according to the criteria; however, the morphology of individual lesions is compatible with LCV arranged in an annular pattern, and as a result of this together with the histologic and DIF findings diagnostic for LCV, we believe that our patient should be classified as having annular LCV. The presence of extensive generalized erythematous maculopapular rash on our patient’s trunk and extremities might have obscured the initiation of the LCV lesions; hence, onset of annular LCV was imprecise. The day after resolution of the maculopapular rash, multiple, well-defined, non-blanchable, erythematous-to-purplish macules and papules, some of which showed an annular arrangement, could be clearly noticeable.

Annular LCV is an uncommonly reported clinical variant of LCV. Annular LCV has been linked with systemic diseases such as sarcoidosis, ulcerative colitis, cryoglobulinemia associated with hepatitis B, Sjögren’s syndrome, cervical cancer, lymphoma, and monoclonal and polyclonal gammopathies [1,2]. Annular LCV has also been linked to pregnancy, chlorzoxazone, sorafenib and amlodipine besylate [7-10]. Amlodipine was not the culprit drug for the development of LCV in our patient’s case because he had been administered it for many years. Moreover, his condition resolved despite continuation of amlodipine. An extensive review of the literature revealed no previous case reports of annular LCV associated with anti-tuberculosis drugs.

## Conclusions

In our patient’s case, the histology and resolution after drug discontinuation suggest that anti-tuberculosis pharmaceuticals may have been the offending drugs that caused annular LCV. LCV should be considered in the differential diagnosis of annular non-blanchable macules and papules. Although rarely seen, anti-tuberculosis drugs should be considered potential causes of drug-induced annular LCV.

### Consent

Written informed consent was obtained from the patient for publication of this manuscript and any accompanying images. A copy of the written consent is available for review by the Editor-in-Chief of this journal.

## Competing interests

The authors declare that they have no competing interests.

## Authors’ contributions

WR, KT and KC prepared the text and collected all the medical data. WR and KC reviewed the literature, provided suitable references and assisted with the draft version of the paper. WR and KC reviewed and interpreted the histopathology findings and prepared them for the manuscript. WR, KT and KC reviewed the paper and revised the final version. All authors read and approved the final manuscript.
